# The Newly Developed CRF_1_-Receptor Antagonists, NGD 98-2 and NGD 9002, Suppress Acute Stress-Induced Stimulation of Colonic Motor Function and Visceral Hypersensitivity in Rats

**DOI:** 10.1371/journal.pone.0073749

**Published:** 2013-09-06

**Authors:** Mulugeta Million, Jing-Fang Zhao, Andrew Luckey, József Czimmer, George D. Maynard, John Kehne, Diane C. Hoffman, Yvette Taché

**Affiliations:** 1 CURE/Digestive Diseases Research Center, Department of Medicine, Division of Digestive Diseases, The David Geffen School of Medicine, University of California Los Angeles, Los Angeles, California, United States of America; 2 Oppenheimer Family Center for Neurobiology of Stress, Department of Medicine, Division of Digestive Diseases, The David Geffen School of Medicine, University of California Los Angeles, Los Angeles, California, United States of America; 3 VA Greater Los Angeles Healthcare System, Los Angeles, California, United States of America; 4 Neurogen Corporation, Branford, Connecticut, United States of America; University of Arizona, United States of America

## Abstract

Corticotropin releasing factor receptor 1 (CRF_1_) is the key receptor that mediates stress-related body responses. However to date there are no CRF_1_ antagonists that have shown clinical efficacy in stress-related diseases. We investigated the inhibitory effects of a new generation, topology 2 selective CRF_1_ antagonists, NGD 98-2 and NGD 9002 on exogenous and endogenous CRF-induced stimulation of colonic function and visceral hypersensitivity to colorectal distension (CRD) in conscious rats. CRF_1_ antagonists or vehicle were administered orogastrically (og) or subcutaneously (sc) before either intracerebroventricular (icv) or intraperitoneal (ip) injection of CRF (10 µg/kg), exposure to water avoidance stress (WAS, 60 min) or repeated CRD (60 mmHg twice, 10 min on/off at a 30 min interval). Fecal pellet output (FPO), diarrhea and visceromotor responses were monitored. In vehicle (og)-pretreated rats, icv CRF stimulated FPO and induced diarrhea in >50% of rats. NGD 98-2 or NGD 9002 (3, 10 and 30 mg/kg, og) reduced the CRF-induced FPO response with an inhibitory IC_50_ of 15.7 and 4.3 mg/kg respectively. At the highest dose, og NGD 98-2 or NGD 9002 blocked icv CRF-induced FPO by 67–87% and decreased WAS-induced-FPO by 23–53%. When administered sc, NGD 98-2 or NGD 9002 (30 mg/kg) inhibited icv and ip CRF-induced-FPO. The antagonists also prevented the development of nociceptive hyper-responsivity to repeated CRD. These data demonstrate that topology 2 CRF_1_ antagonists, NGD 98-2 and NGD 9002, administered orally, prevented icv CRF-induced colonic secretomotor stimulation, reduced acute WAS-induced defecation and blocked the induction of visceral sensitization to repeated CRD.

## Introduction

Corticotropin releasing factor (CRF), a 41-amino acid peptide originally isolated from ovine brain extract, is the principal mediator of the hypothalamic-pituitary-adrenal (HPA) stress–response [1], [2] CRF exerts its biological functions by activating two classes of B subfamily G-protein coupled receptors, CRF_1_ and CRF_2_ receptors [3]. Activation of brain CRF_1_ signaling by CRF peptides plays a pivotal role in the behavioral, endocrine, immune, autonomic, and visceral responses to stress [2], [4]–[6]. One of the bodily systems susceptible to stress and stress-related peptides is the gastrointestinal tract [7]. Specifically, acute stressors and CRF injected into the brain or the periphery induces a rapid onset stimulation of colonic motor function in rodents, a response that is largely mediated by activating CRF_1_ receptors in both the brain and the colon and reproducing symptoms of irritable bowel syndrome (IBS) with diarrhea (IBS-D) [8], [9].

Preclinical and early clinical studies support the possibilities that pharmacological interventions targeting CRF_1_ signaling may have potential therapeutic benefits in alleviating stress sensitive disorders [10], [11]. For instance, the peptide CRF receptor antagonist, α–CRF_9–41_, injected into the circulation alleviates symptoms in a subclass of IBS patients [12]. As peptide compounds are less desirable in drug development, non-peptide small molecule CRF receptor antagonists are being developed to treat anxiety, depression, alcoholism, drug relapse and stress-related gastrointestinal diseases [10], [13]–[15]. Progress in the therapeutic use of non-peptide CRF_1_ antagonists, however, has been slow and largely disappointing due in part to the lack of consistency in their efficacy. For instance, chronic administration of a selective CRF_1_ antagonist, R121919/NBI 30775, showed anxiolytic and antidepressant effects in the first open-label clinical study in patients with major depressive episodes [16]. NBI-34041 showed efficacy against the Trier social stress-induced endocrine response in placebo-controlled phase I and II clinical trials performed in healthy subjects [11]. There is also preliminary evidence that R317573 exerts anxiolytic effects in healthy subjects subjected to 7.5% carbon monoxide inhalation, an experimental model of anxiety [17]. Similarly, in a recent randomized, double-blind, placebo-controlled study, the selective CRF_1_ antagonist GSK-GW876008 decreased brain regional activity associated with the emotional-arousal network during expectation of abdominal pain in IBS patients [14]. On the other hand, the CRF_1_ antagonists, CP-316,311, showed no effect against depression in a 6-week randomized, placebo-controlled trial [18] and pexacerfont did not demonstrate efficacy compared to placebo for the treatment of generalized anxiety disorders in a multi-center clinical trial [19]. With regard to IBS, a double blind placebo-controlled clinical report showed the lack of effect of the CRF_1_ selective antagonist BMS-562086 in ameliorating gastrointestinal symptoms in IBS-D patients [15].

Enthusiasm for the first generation of selective non-peptide CRF_1_ antagonists, including CP-154,526 [20] and SSR125543A [21] was dampened by their pharmacokinetic properties. Overall the CRF_1_ antagonists, with demonstrated high selectivity and potency in *in vitro* biological tests and preclinical assays, were highly lipophilic and hence less attractive for therapeutic use due to the potential risk of elevated tissue accumulation and prolonged half life [22], [23]. Furthermore, the improvements in decreasing lipophilicity are not necessarily translated to higher oral bioavailability. Thus, to date there are very few CRF_1_ receptor antagonists with high oral bioavailability and desirable pharmacokinetic profile.

Recently, we have developed and described a new generation of topology 2 selective CRF_1_ antagonists with pyrazine cores, namely NGD 98-2 *(5-(2-Methoxy-4-trifluoromethoxyphenyl)-[N-(1-ethyl)propyl]-3-methoxy-6-methylpyrazine-2-amine tosylate)* and NGD 9002 *(5-(6-isopropyl-2-methylaminopyridin-3-yl)-[N-(1-ethyl)propyl]-3-methoxy-6-methylpyrazine-2-amine hydrochloride).* These compounds displayed K(i) values below 10 nM with acceptable properties and minimal toxicity [24], [25]. In vivo, oral pretreatment with NGD 98-2 prevented intracerebroventricular (icv) CRF-induced increased locomotor activity and acute restraint-stress-induced elevation of plasma ACTH levels in rats [24].

In this study, we examined the antagonist action of NGD 98-2 and NGD 9002 on CRF-induced IBS-D-like symptoms, namely altered colonic motor function and visceral nociceptive hyper-responsiveness to colorectal distention (CRD) in conscious rats. We first delineated the doses at which orogastric (og) and subcutaneous (sc) administration of these compounds will antagonize CRF injected icv or intraperitoneally (ip)-induced stimulation of colonic propulsive motor function and diarrhea [26], [27]. We then used the maximal effective oral dose of NGD 98-2 and NGD 9002 to assess whether this will counteract defecation induced by water avoidance stress (WAS) and the development of visceral hypersensitivity induced by repeated tonic CRD in rats known to involve activation of CRF_1_ signaling [27]–[29].

## Materials and Methods

### 1. Animals

Adult male Sprague-Dawley rats (Harlan, San Diego, California, USA) weighing 280–320 g were housed in group cages with free access to Purina rat chow and tap water. Animals were quarantined under controlled conditions of illumination (12 h light/dark cycle; lights on 06∶00 h), temperature, and humidity for at least one week. Experiments started between 9 am and 10 am in non-fasted rats unless otherwise stated. Experimental protocols were approved by the Animal Care Committee of the Veteran Affairs Greater Los Angeles Healthcare System (#06-069-02) and the UCLA Animal Research Committee UCLA (ARC #2002-042).

### 2. Substances

**Figure 1:**
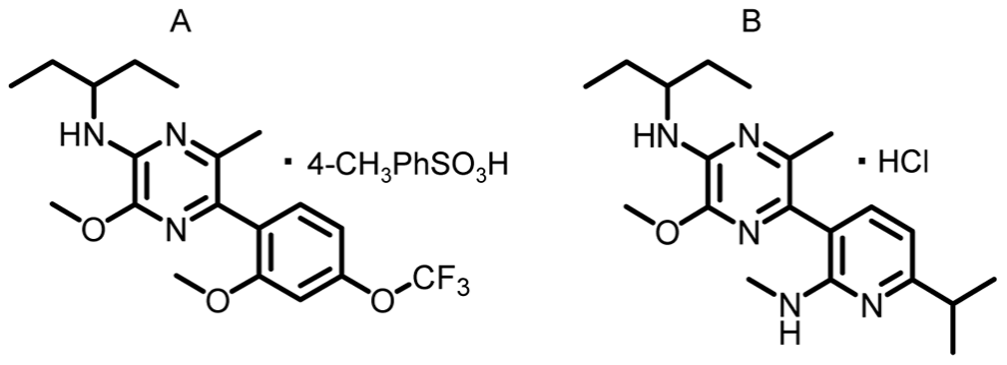
Chemical structures of A = NGD 98-2∶5-(2-Methoxy-4-trifluoromethoxyphenyl)-[N-(1-ethyl)propyl]-3-methoxy-6-methylpyrazine-2-amine tosylate. B = NGD 9002∶5-(6-isopropyl-2-methylaminopyridin-3-yl)-[N-(1-ethyl)propyl]-3-methoxy-6-methylpyrazine-2-amine hydrochloride.

NGD 98-2 (5-(2-methoxy-4-trifluoromethoxyphenyl)-[N-(1-ethyl)propyl]-3-methoxy-6-methylpyrazine-2-amine tosylate) (Fig. 1A) and NGD 9002 (5-(6-isopropyl-2-methylaminopyridin-3-yl)-[N-(1-ethyl)propyl]-3-methoxy-6-methylpyrazine-2-amine hydrochloride) (Fig. 1B) were synthesized at Neurogen Corporation (Branford, CT, USA) [24]. For oral preparation, a day before the experiment, compounds were sonicated and suspended in 0.5% methylcellulose in distilled water with 0.1% triacetin (Sigma-Aldrich Co., St. Louis, MO) and placed on a magnetic stir plate overnight. For sc injection, NGD 98-2 and NGD 9002 were dissolved in dimethyl sulfonic acid (DMSO, Sigma-Aldrich):Tween-80:saline (1∶1:8 ratio). Rat/human CRF and astressin (Clayton Foundation Laboratories for Peptide Biology, Salk Institute, La Jolla, CA, USA) were kept at –80^o^ C in powder form and diluted in saline just before administration. The selective non-peptide CRF_1_ antagonist, CP-154,526 (Pfizer, Groton, CT, USA) [30] was diluted in a DMSO:Tween-80-:saline (1∶1:8 ratio) solution as described before [31]. The pH of compound solutions at different concentrations was measured and respective vehicles were adjusted to be at the same pH.

### 3. Substance Administration

The volume of administration varied with the route of delivery: og, 5 or 10 ml/kg; sc, 1.5 ml/kg; ip, 1.0 ml/kg; icv, 10 µl/rat. The og gavage was performed using a stainless steel tubing (Cadence, Inc. Staunton, VA) in lightly hand-restrained rats and the sc injection was made into the loose skin of the back over the shoulders.

The icv injections were performed as in our previous studies [32]. Conscious lightly restrained rats with chronic icv cannula were injected through a 28 ga cannula (Plastics One Inc., Roanoke, VA, USA), 1 mm longer than the guide cannula. The injection cannula was connected to a 50 µl Hamilton syringe by a PE-50 tubing (Intramedic Polyethylene Tubing, Clay Adams, Sparks, MD, USA) filled with distilled water. A small air bubble (1 µl) was drawn at the distal end of the PE-50 tubing to separate the injected solution from the water and for visual monitoring of the icv injection which was performed slowly over a 60-sec period. At the end of experiments, animals were euthanized with sodium pentobarbital overdose followed with bilateral thoracotomy. In icv cannulated rats, the correct location of the cannula into the lateral ventricles was assessed by injecting icv 0.1% toluidine blue (10 µl) and the visualization of dye on the walls of lateral ventricles.

**Figure 2:**
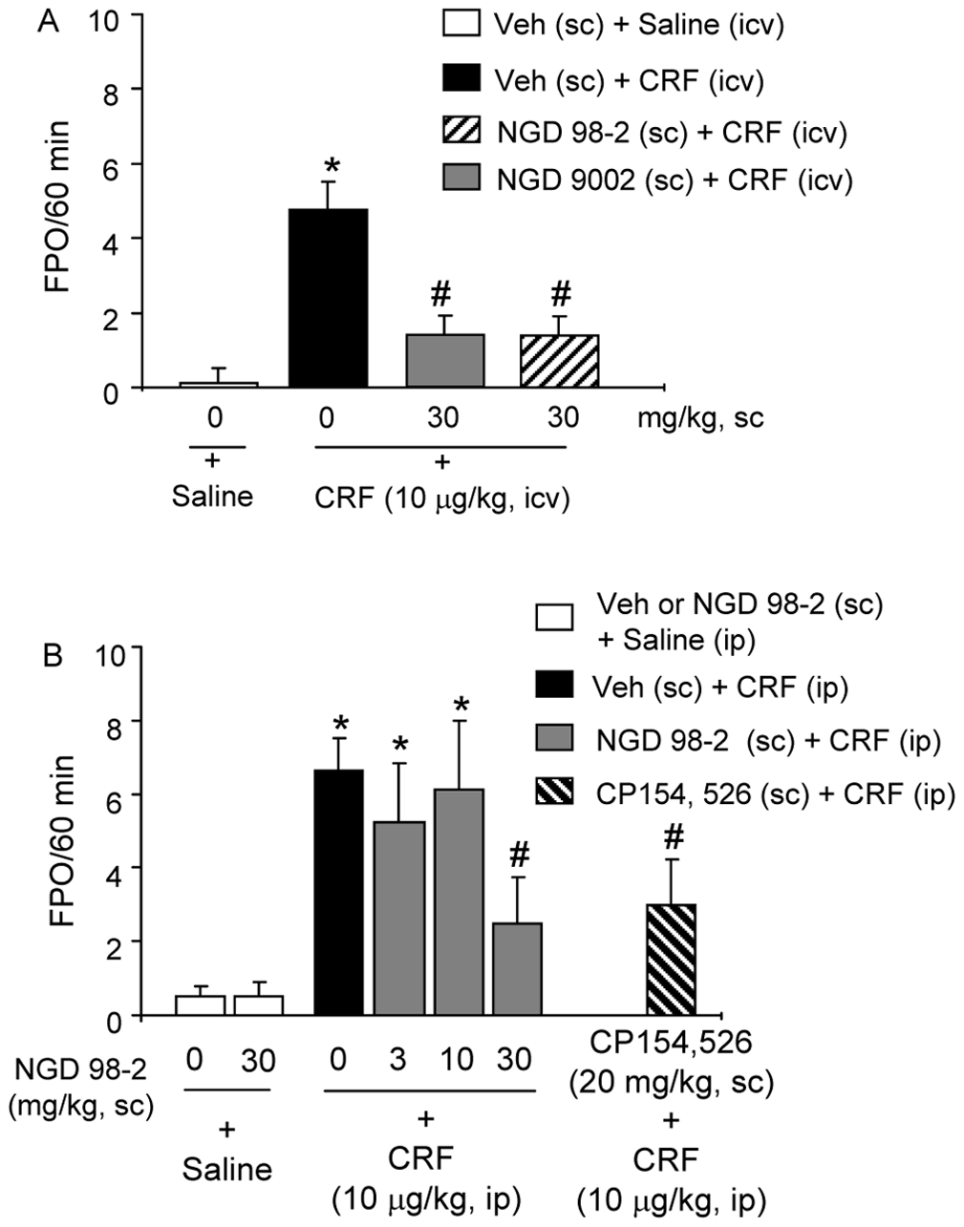
Subcutaneous injection of CRF_1_ antagonists, NGD 9002 or NGD 98-2 prevents central (icv) or systemic (ip) CRF-induced stimulation of propulsive colonic motor function in conscious rats. A: Rats with chronic icv cannula were pretreated sc with vehicle, NGD 98-2 or NGD 9002 and 60 min later were injected icv with saline or CRF and FPO monitored for 60 min. B: Rats were pretreated sc with either vehicle or NGD 98-2 (3, 10, 30 mg/kg) and 60 min later, they were injected ip with CRF or saline. As a positive control, a group of rats was injected sc with a known CRF_1_ antagonist, CP154,526, 60 min prior to ip CRF and FPO monitored for 60 min. Each bar represents the mean and SEM of 8 rats/group. *p<0.05 compared with sc vehicle+icv saline group (A) or vs sc vehicle+ip saline group (B); ^#^p<0.05 compared with sc vehicle+icv CRF (A), or vs sc vehicle+ip CRF B), ANOVA, Student-Newman-Keuls.

**Figure 3:**
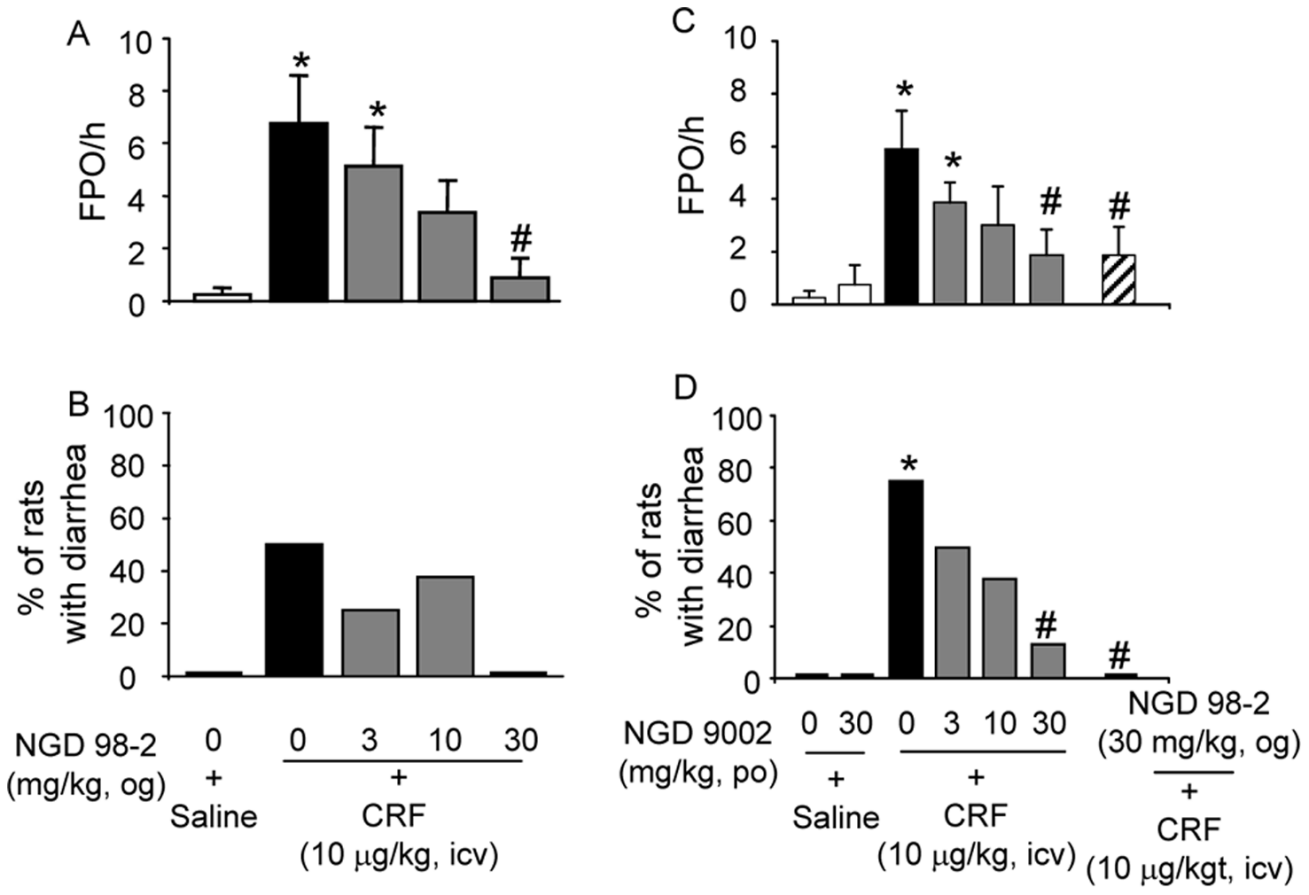
Oro-gastric (og) administration of CRF_1_ antagonist, NGD 98-2 or NGD 9002 blunts icv CRF-induced defecation and diarrhea in rats with chronic icv cannula. NGD 98-2 (3, 10 and 30 mg/kg) or saline was given og 180 min before icv CRF or saline and fecal output (A) and diarrhea (B) were monitored for 1 h post icv injection. NGD 9002 (3, 10, 30 mg/kg) or saline was given 60 min before icv CRF and FPO (C) and diarrhea (D) monitored for 1 h post icv injection. Each bar in A and C represents the mean and SEM while each bar in B and D represents mean % of 8 rats/group. *p<0.05 compared with og vehicle+icv saline group (A-D); ^#^p<0.05 compared with og vehicle+icv CRF (A-D), ANOVA, Student-Newman-Keuls; t-test; Fisher Exact test.

**Figure 4:**
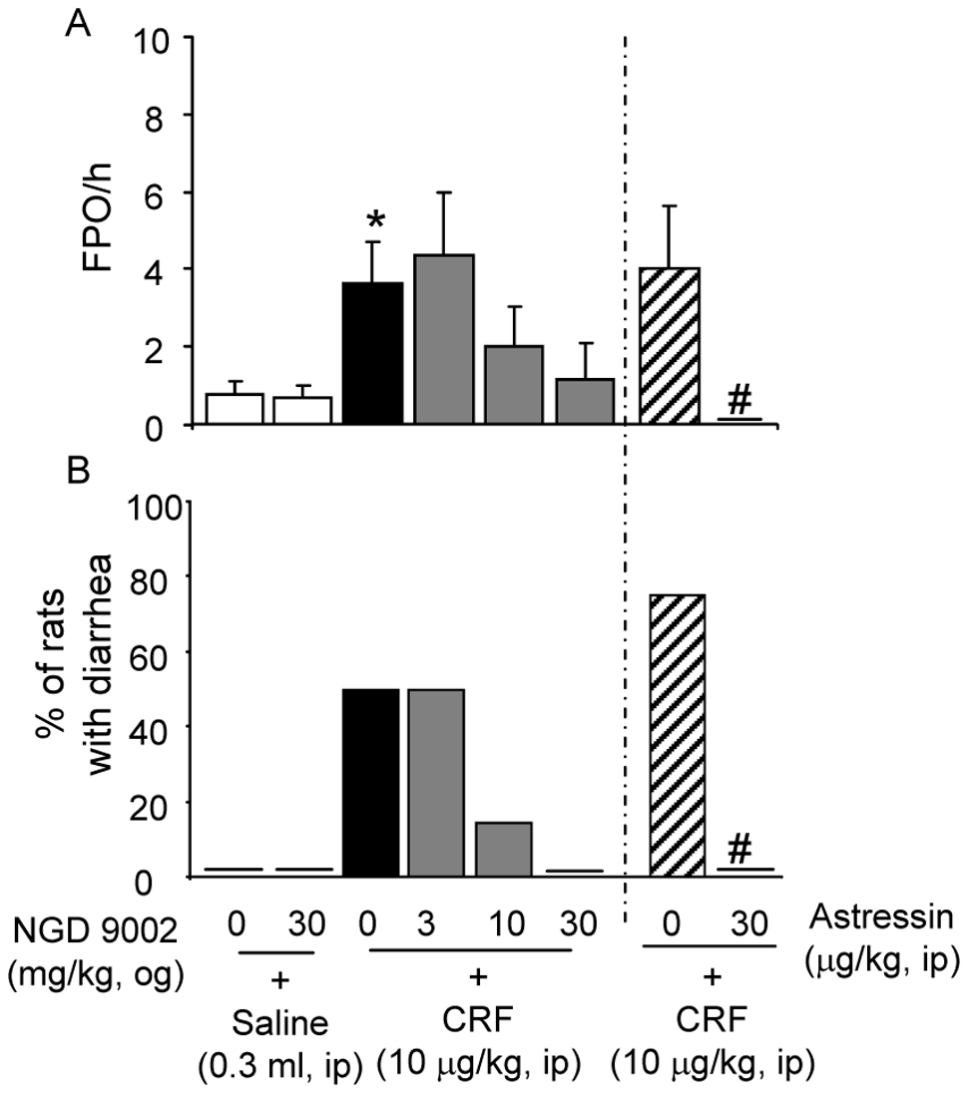
Oro-gastric (og) administration of NGD 9002 exerted a non-significant trend towards reduction of ip CRF-induced FPO and diarrhea in rats. Vehicle or NGD 9002 (3, 10 and 30 mg/kg) was given og 60 min before ip CRF-induced FPO (A) and diarrhea (B) which were monitored for the 60 min post ip injection. As a positive control, group of rats were pretreated with ip astressin (30 µg/kg), a non selective CRF_1_ and CRF_2_ receptor antagonist, just prior to ip CRF. Each bar in A represents the mean and SEM of FPO while in B they represent the mean % of 8 rats/group. *p<0.05 vs saline+vehicle or saline+NGD 9002 (30 mg/kg); ^#^p<0.05 compared with the corresponding ip astressin+ip CRF. ANOVA, Student-Newman-Keuls; t-test; Fisher Exact test.

**Figure 5:**
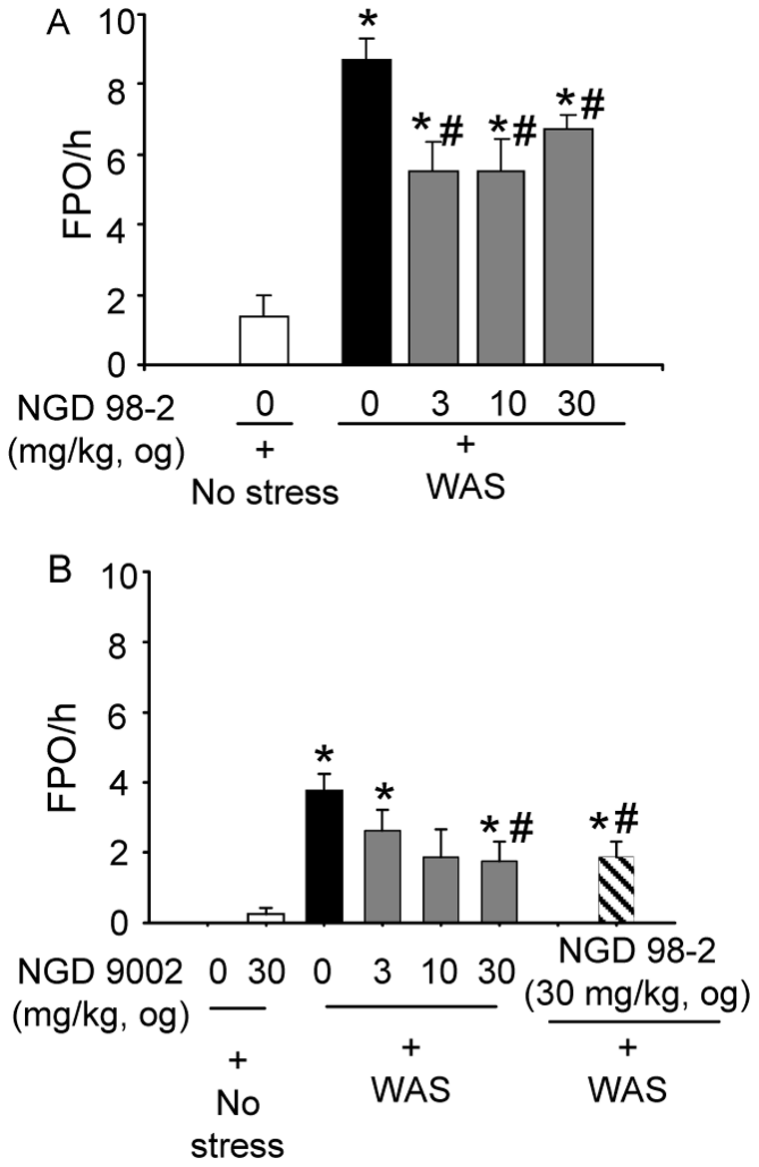
Oro-gastric (og) administration of NGD CRF_1_ antagonist, NGD 98-2 or NGD 9002 reduces acute water avoidance stress (WAS)-induced stimulation of colonic propulsive motor function in rats. Vehicle or NGD 98-2 (A) or NGD 9002 (B) at 3, 10 and 30 mg/kg was administered po and 180 min later (for NGD 98-2 group) or 60 min later (for NGD 9002 group), rats were exposed to WAS for 60 min. FPO was monitored during the 60 min stress session. Each bar represents the mean and SEM of 8 rats/group. *p<0.05 compared with vehicle og+no stress; ^#^p<0.05 compared with vehicle og+WAS, ANOVA, Student-Newman-Keuls.

**Figure 6:**
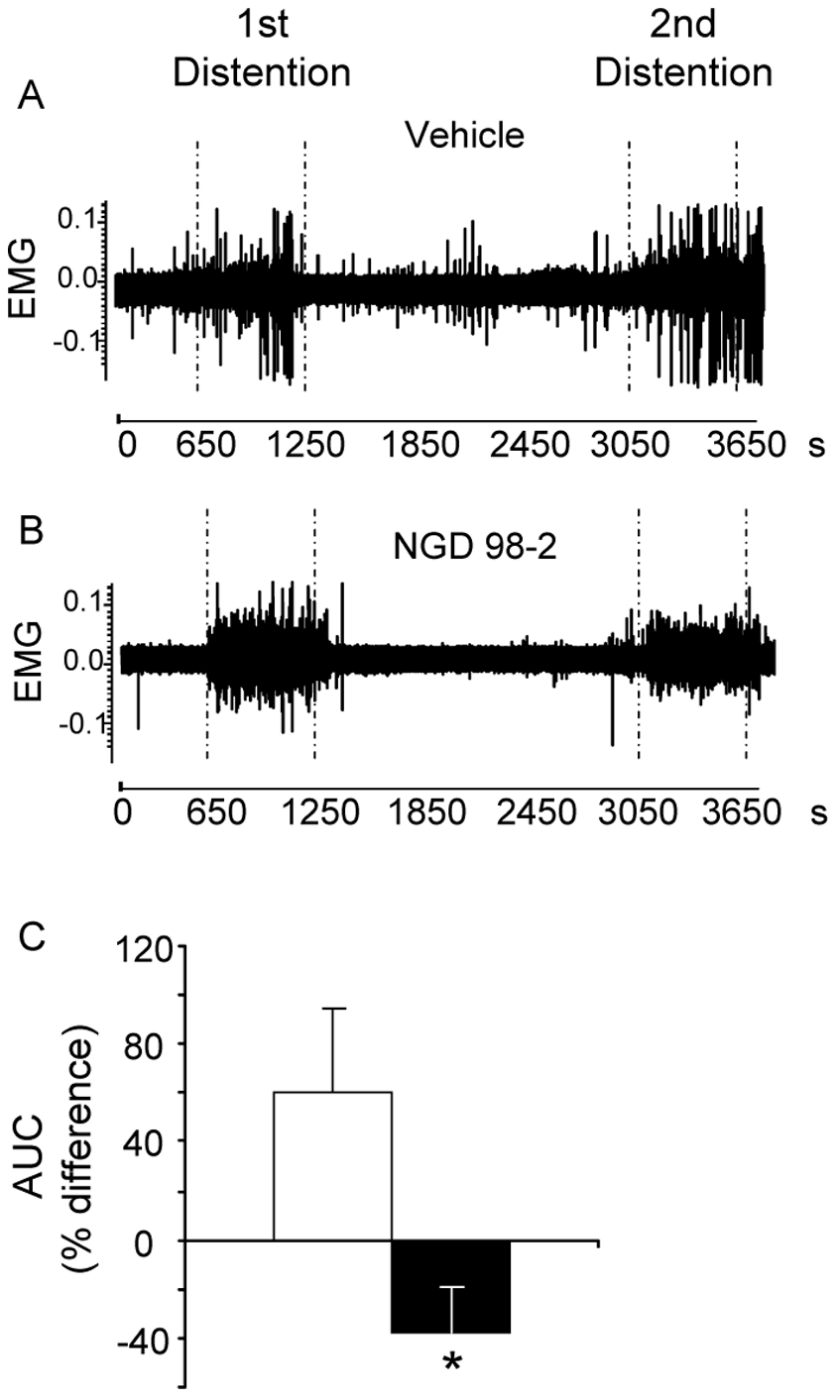
Oro-gastric (og) pretreatment with NGD 98-2 blunts repeated tonic colorectal distention (CRD)-induced visceral sensitization response in conscious rats. Representative trace of abdominal muscle electromyogram (EMG) of rats pretreated with vehicle (A) or NGD 9002 (B). Percent difference in the area under the curve of contraction (AUC) between the 1^st^ and the 2^nd^ dissentions is shown in C. Rats were chronically implanted with abdominal electrodes and ∼10 days later were pretreated og with vehicle or NGD 98-2 (30 mg/kg). After 30 min of habituation and 10 min basal recording, rats were submitted to the first CRD (10 minutes at 60 mm Hg) followed by a 30 min rest and a 2^nd^ 10 min distention at 60 mmHg. Values are mean and SEM of percent differences between the first and second responses to CRDs of 9–10 rats/group. *p<0.05 versus vehicle, t-test or ANOVA, Student-Newman-Keuls.

**Figure 7:**
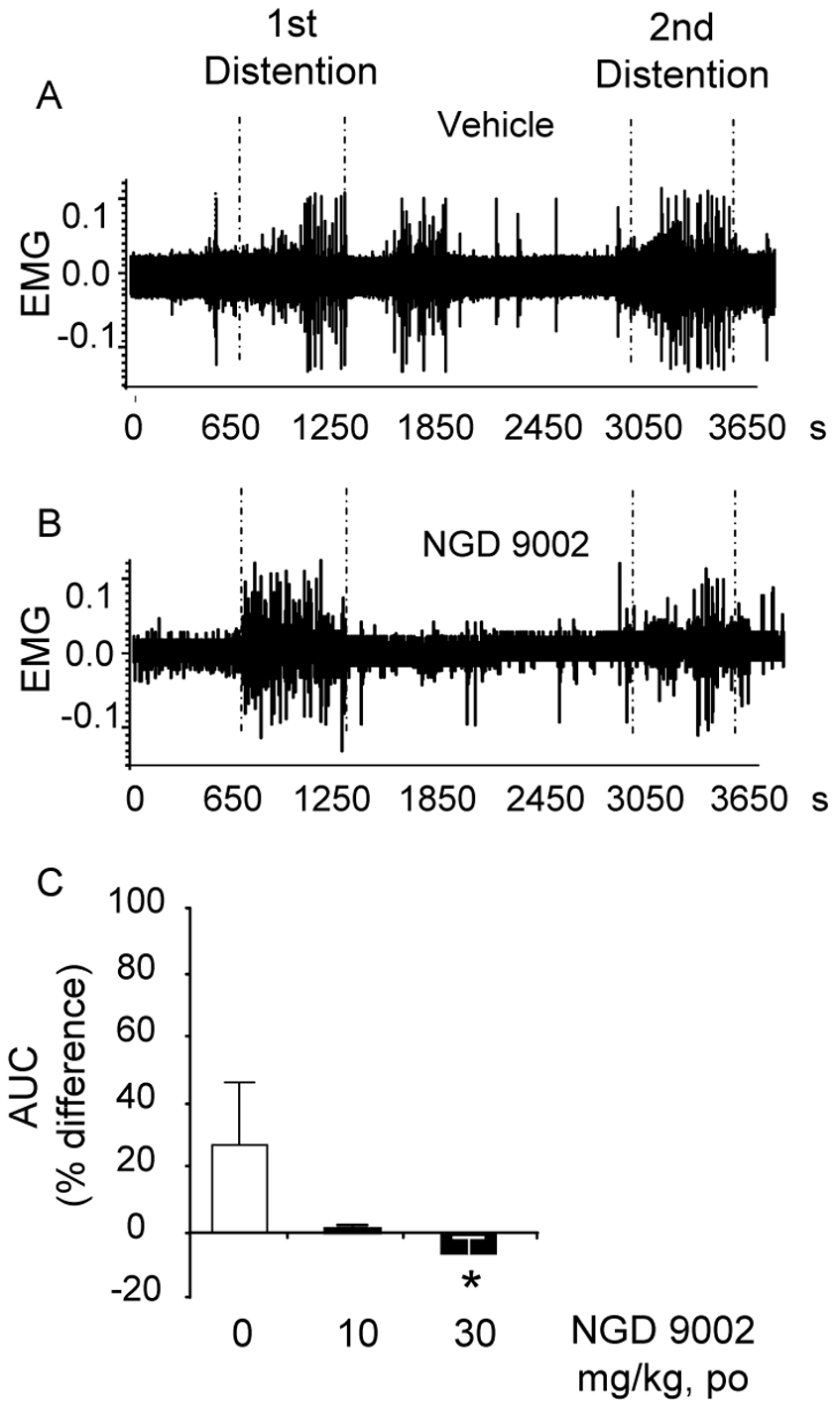
Oro-gastric (og) pretreatment with NGD 9002 blunts repeated tonic colorectal distention (CRD)-induced visceral sensitization response in conscious rats. Rats were chronically implanted with abdominal electrodes and ∼10 days later were pretreated og with vehicle or NGD 9002 (0, 10 or 30 mg/kg). After 30 min of habituation and 10 min basal recording, rats were submitted to the 1^st^ CRD (10 minutes at 60 mm Hg) followed by a 30 min rest and a 2^nd^ 10 min distention at 60 mmHg. Representative trace of abdominal muscle electromyogram (EMG) of rats pretreated with vehicle (A) or NGD 9002 (B). Percent difference in the area under the curve of contraction (AUC) between the 1^st^ and the 2^nd^ dissentions in saline and NGD 9002 treated rats is shown in C. Values are mean and SEM of percent differences between the first and second responses to CRDs of 8–22 rats/group. *p<0.05 versus vehicle, t-test or ANOVA, Student-Newman-Keuls.

The regimens of compound administration were as follow: the sc injection of NGD 98-2 was performed 60 min before icv or ip CRF (Fig. 2A and B) and og administration, 180 min before icv CRF (Fig. 3) or WAS. Oro-gastric or sc NGD 9002 was given 60 min before icv (Fig. 2 A) or ip CRF (Fig. 4) or WAS (Fig. 5). For CRD-induced visceral nociceptive responsivity, both NGD 98-2 and NGD 9002 were given og 40 min prior to the 1st CRD (Fig 6 and 7). These dosing regimens were based on our previous report showing that NGD 98-2 given orally 180 min before icv CRF prevents the CRF-induced increased locomotor activity or restraint stress-induced elevation of plasma ACTH levels [24] as well as pilot studies to assess optimal inhibitory effect on icv CRF-induced defecation.

### 4. Surgeries

#### 4.1. Intracerebroventricular cannulation

ICV cannulation was performed as previously reported [32]. Rats were anesthetized with an ip injection of a mixture of ketamine (75 mg/kg; Fort Dodge Laboratories, Fort Dodge, IA, USA) and xylazine (5 mg/kg; Mobay Corporation, Shawnee, KS, USA). A chronic guide cannula (22 ga, Plastic One Products) was implanted into the right lateral brain ventricle according to coordinates from Paxinos and Watson [33] (mm from bregma: antero–posterior, −0.8; lateral, −1.5; dorsoventral, −3.5). The guide cannula was maintained in place by dental cement anchored by four stainless steel jewelry screws fixed to the skull. The cannula was capped with a dummy cannula for protection. Following icv cannulation, rats were housed singly and allowed to recover for at least 7-10 days during which they were trained to the experimental conditions of icv injection by handling them for 5 min per day for at least 3 consecutive days.

#### 4.2. Abdominal muscle electrodes implantation

The implantation of electrodes was performed in rats anesthetized with an ip injection of a mixture of ketamine (75 mg/kg) and xylazine (5 mg/kg) as detailed in previous studies [34]. Under aseptic conditions, a 2–3 cm incision was made near the lower abdomen along the mid-line. The abdominal muscle layers and the peritoneum were opened and a group of 3 teflon coated silver electrodes were fixed in triangular pattern (5 mm apart) on the left side of the abdominal muscle 1–2 cm lateral to the mid line. The other end of the electrodes was fitted in a small plastic casing, which served as a jack to connect the electrodes to the recording device. The connecting side of the jack was then exteriorized on the right side of the flank (2–3 cm lateral to the mid-line) through a small (3 mm diameter) opening across the abdominal wall and the skin. The base of the jack was secured in place by suturing it onto the peritoneal side of the abdominal muscle. Rats were allowed to recover from surgery for 10–15 days.

### 5. Procedures

#### 5.1. Water avoidance stress

The WAS was performed as described before [35] by placing the rat on a small cubic pedestal (8 cm height, 6 cm wide) positioned in the center of a plastic cage filled with room temperature water up to 7 cm height of the pedestal. To avoid contact with the water, the rat remains on the pedestal platform for the experimental period.

#### 5.2. Measurements of abdominal contractions to colorectal distention

Rats chronically fitted with electrodes on abdominal muscles were trained to the experimental conditions by placing them in Bollman cages for 2–3 h/day for at least 3 consecutive days before the study. On the day of the experiment, rats were briefly anaesthetized with isoflurane (3% in O_2_), and a 6 cm long plastic balloon tied around an Intramedic PE-100 tubing (Becton Dickinson, Franklin Lakes, NJ, USA) was inserted intra-anally with the distal end positioned 1 cm proximal to the anus. The CRD in awake rats results in contractions of the abdominal and hind limb musculature and this visceromotor response (VMR) is validated as a quantitative measure of visceral hypersensitivity [36]. The protocol of CRD was similar to our previous studies showing the induction of visceral hypersensitivity [29], [37]. CRD entailed inflating the CRD balloon with a barostat (Distender II, J&J Inc., Toronto, ON, Canada) at 60 mmHg twice for 10 min with a 30 min rest interval. The VMR to CRD was recorded as electromyography (EMG) signals and acquired using a Micro1401 A/D interface (Cambridge Electronic Design, Ltd, Cambridge, UK) connected to a Pentium IV class computer running Spike 2 data acquisition software. EMG signals were amplified, filtered (x10000, 300–5000 Hz), digitized, and rectified as detailed previously [38]. The basal area under the curve (AUC) of abdominal contractions recorded from EMG was calculated as the area under the rectified EMG signal trace for the 10 min period immediately preceding the first 10 min CRD. The AUC values of the EMG during the first and second distensions were computed and basal AUC subtracted to obtain the net AUC in response to CRD as described [37]. AUC of contractions in response to each 10 min distention was compared to each other. From these values, the percent change in AUC [ΔAUC (%)] was calculated by taking the difference between the 1^st^ and 2^nd^ distention AUC responses and dividing by the 1^st^ AUC and multiplying by 100.

#### 5.3. Colonic motor function measurements

Defecation was monitored as described previously [31] by counting the number of fecal pellets excreted every 15 min for up to 2 h. The incidence of diarrhea was assessed for the 2-h period post CRF injection as percent of rats that developed one loose-watery stool or more from the total number of treated rats.

### 6. Experimental Protocols

All the experiments started between 9–10 am and were conducted in non-fasted conscious rats trained to the experimental conditions. Unless otherwise stated, antagonists were administered following a regimen of 60 min pretreatment period.

#### 6.1. Effect of NGD 98-2 or NGD 9002 injected subcutaneously on intracerebroventricular or intraperitoneal CRF-induced stimulation of colonic secretomotor function

Groups of rats (n = 8/group) were injected sc with either vehicle (DMSO:Tween 80:saline in 1∶1:8 ratio), NGD 98-2 or NGD 9002 (30 mg/kg) before icv CRF (10 µg/kg) or saline. Fecal pellet output (FPO) was then monitored for 60 min post icv injection.

In a separate experiment, rats (n = 8/group) were injected sc with either vehicle, NGD 98-2 (3, 10 or 30 mg/kg) or CP 154,526 (20 mg/kg) followed with ip saline or CRF (10 µg/kg) and fecal pellet and diarrhea responses were monitored for 60 min post ip injection. We previously reported the efficacy of CP 154,526 (20 mg/kg) against ip CRF (10 µg/kg) -induced stimulation of defecation in rats [39] and this CRF_1_ antagonist at such a dose was used as a positive control.

#### 6.2. Dose-related effect of NGD 98-2 or NGD 9002 administered orogastrically on CRF or water avoidance stress-induced colonic motor response

For intracerebroventricular CRF, saline or CRF (10 µg/kg) was injected icv in chronically cannulated rats, 180 min or 60 min after og administration of vehicle (0.5% methylcellulose in distilled water with 0.1% triacetin), NGD 98-2 (3, 10 or 30 mg/kg) or NGD 9002 (3, 10 or 30 mg/kg) (n = 8/group). The FPO and diarrhea responses were monitored for 60 or 120 min post icv injection.

For intraperitoneal CRF, in a separate set of experiments, NGD 9002 (3, 10 or 30 mg/kg) or its vehicle was administered orogastrically before ip injection of CRF (10 µg/kg) or saline (n = 8/group). The defecation response was monitored for 60–120 min. The peptide CRF antagonist astressin injected ip (30 µg/kg) immediately before ip CRF (10 µg/kg) was used as positive control. Astressin under these conditions is known to block ip CRF-mediated colonic response [31].

For water avoidance stress, rats were pretreated orogastrically with either vehicle (0.5% methylcellulose in distilled water with 0.1% triacetin) or NGD 98-2 (3, 10 or 30 mg/kg) or NGD 9002 (3, 10 or 30 mg/kg) and 180 min later (for NGD 98-2) or 60 min later (for NGD 9002), rats (n = 8/group) were either left undisturbed in their home cage (no stress) or exposed to WAS for 60 min.

#### 6.3. Effect of orally-administered NGD 98-2 or NGD 9002 on repeated colorectal distention-induced visceral nociceptive sensitization

After rectocolonic positioning of the balloon and recovery from the short anesthesia, rats were administered og with NGD 9002 (10 or 30 mg/kg, n = 8 or 17/group) or NGD 98-2 (30 mg/kg, n = 10) or their vehicle (0.5% methylcellulose/0.1% triacetin, n = 22) and placed in Bollman cages. After a 30-min stabilization period and 10-min baseline recording, all groups were submitted to isobaric CRD (60 mm Hg for 10 min twice, with a 30 min rest interval) using a barostat. The abdominal contraction responses to the 1^st^ and 2^nd^ distention were compared within and between groups.

### 7. Statistical Analysis

Values are expressed as mean and standard error of the mean or mean % difference. The FPO response to different treatments or doses was compared using a one-way analysis of variance (ANOVA). Comparison between groups on diarrhea incidence (%) was made using Fisher’s exact test. Two way ANOVA was used, to compare the two factor effects of NGD 9002 and NGD 98-2 (0, 3, 10, 30 mg/kg) on CRF or WAS-induced FPO responses. Similarly, where appropriate, IC_50_ of NGD compounds was calculated using Graphpad Prism Software (GraphPad Software, Inc. La Jolla, CA). The AUC of abdominal contraction response to the 1^st^ vs 2^nd^ distension within a group were compared using before and after paired t-test or one-way repeated measures ANOVA and percent differences using t-test or ANOVA. For pair wise multiple comparisons, Student-Newman-Keuls Method or Fisher LSD test was used. P<0.05 was considered as a significant difference.

## Results

### 1. NGD 98-2 or NGD 9002 Injected Subcutaneously Inhibits icv and ip CRF-induced Stimulation of Propulsive Colonic Motor Function in Rats

In chronic icv cannulated rats, CRF (10 µg/kg, icv) injected 60 min after vehicle increased FPO compared to icv saline (number/60 min: 4.8±0.8 vs 0.0±0.0; p<0.05; n = 8/group). Pretreatment (30 mg/kg sc, −60 min) with NGD 98-2 or NGD 9002 inhibited icv CRF-induced FPO by 71% for each compound (1.4±0.6 and 1.4±0.6 pellet/60 min respectively, p<0.05, n = 8/group) (Fig. 2A).

Similarly, in vehicle pretreated (sc, −60 min) rats, ip injection of CRF (10 µg/kg), significantly stimulated FPO compared with saline (number/60 min 6.6±0.9 vs 0.5±0.3; p<0.05; n = 8/group). Pretreatment (30 mg/kg sc, −60 min) with NGD 98-2 inhibited significantly the colonic response to ip CRF by 59% (2.5±1.2 pellet/1h; p<0.05, n = 8/group) (Fig. 2B) while at lower doses (3 and 10 mg/kg, sc), NGD 98-2 had no significant effect (5.3±1.6 and 6.1±1.9 pellet/1h, respectively; p>0.05, n = 8/group, Fig. 2B). Rats pretreated (sc, −60 min) with the highest dose of NGD 98-2 (30 mg/kg) had a similar reduction of ip CRF-induced stimulation of FPO as that of the known selective CRF_1_ antagonist, CP154,526 (20 mg/kg, sc −60 min) (Fig. 2B). NGD 98-2 at 30 mg/kg alone had no effect on FPO (Fig. 2B).

### 2. NGD 98-2 or NGD 9002 given Orally Decreases Dose-dependently icv-CRF-Induced Stimulation of Propulsive Colonic Motor Function

In vehicle-pretreated (og, −180 min) rats with a chronic icv cannula, CRF (10 µg/kg, icv) significantly stimulated FPO compared with the og vehicle+icv saline group during the 60 min period post icv injection (6.8±1.8 vs 0.5±0.3 pellets/60 min; n = 8/group, p<0.05, Fig. 3A). In addition, 50% of the icv CRF-injected rats developed diarrhea (P = 0.07). Pretreatment (og, −180 min) with NGD 98-2 (3, 10, and 30 mg/kg, n = 8 for each dose), dose-dependently inhibited the FPO responses to icv CRF vs vehicle (pellets/60 min: 5.1±1.5, 3.4±1.2, and 0.9±0.7 respectively vs 6.8±1.8, p<0.05 at the highest dose) with an IC_50_ of 15.7 mg/kg (Fig. 3A). There was also a non-significant trend towards reduction of the 50% incidence of diarrhea in the og vehicle+icv CRF group to 25%, 37.5%, and 0% in response to og pretreatment with NGD 98-2 at 3, 10 and 30 mg/kg, respectively (Fig. 3B). Likewise, pretreatment (-60 min) with NGD 9002 (3, 10, and 30 mg/kg, og, n = 8 for each dose) reduced the icv CRF-stimulated FPO (5.9±1.5 vs iccv saline 0.3±0.3 number/60 min, p<0.05) to 3.9±0.7, 3.0±1.4 and 1.9±0.9 number/60 min respectively (p<0.05 at the highest dose, Fig. 3C) with an IC_50_ of 4.3 mg/kg. Orogastric NGD 9002 also reduced dose-dependently the icv CRF-induced 75% incidence of diarrhea reaching significance at the 30 mg/kg dose (12.5%, p<0.05; Fig. 3D). NGD 98-2 (30 mg/kg, og), used as a positive control in this particular experiment, completely prevented the incidence of icv CRF-induced diarrhea (0%, p<0.05; Fig. 3D). In the absence of CRF, NGD 9002 alone (30 mg/kg, og) had no effect on FPO or diarrhea (Fig. 3C–D). A two-way ANOVA to assess drug (NGD 98-2 and NGD 9002) and dose (0, 3, 10 and 30 mg/kg) interactions showed a significant main effect of drug (p<0.01) as well as dose (p<0.01) on icv CRF-induced FPO response and no significant interaction between drug type and dose (P = 0.396).

### 3. NGD 9002 Administered Orogastrically and Astressin Intraperioneally Reduce ip-CRF-induced Colonic Responses

In vehicle-pretreated (-60 min) rats, CRF injected ip (10 µg/kg) stimulated fecal output (3.6±1.0 vs 0.8±0.3 pellets/60 min; p = 0.05; n = 8/group, Fig. 4A). Compared to vehicle, CRF injection induced also diarrhea in 50% of rats (0% vs 50%, p = 0.07, Fig. 4B). Pretreatment (-60 min) with NGD 9002 (3, 10 and 30 mg/kg, og, n = 8 for each dose) induced a trend to reduce ip-CRF stimulated FPO which did not reach statistical significance (2.0±1.0 and 1.1±0.9 pellet/60 min at 10 and 30 mg/kg, respectively; 8/group, p = >0.05, Fig. 4A). Similarly, compared to the vehicle group, 50% of the vehicle+ip CRF or NGD 9002 (3 mg/kg)+ip CRF-treated rats had diarrhea (0% vs 50%, p = 0.07, n = 8/group, Fig 4B). The diarrhea response was 14.3% and 0% in NGD 9002-treated rats at 10 and 30 mg/kg respectively (n = 8 for each dose, Fig. 4B). Compared with rats pretreated with ip saline, astressin (30 µg/kg, ip) abolished both the FPO (4.0±1.3 vs 0 pellet/60 min, p<0.05, Fig. 4A) and diarrhea (75% vs 0%, p<0.05, Fig. 4B) responses to ip CRF.

### 4. NGD 98-2 or NGD 9002 given Orogastrically Decreases Acute Water Avoidance Stress-induced Colonic Motor Response in Rats

The exposure to WAS for 60 min stimulated FPO compared with non-stressed rats maintained in their home cage (8.8±0.6 vs 1.4±0.6 pellet/60 min, p<0.05, n = 8/group, Fig. 5A). Pretreatment with NGD 98-2 (3, 10 and 30 mg/kg, n = 8 for each dose) administered og 180 min before stress attenuated significantly WAS-induced colonic response (8.8±0.6 pellet/60 min) to 5.5±0.8, 5.5±0.9, and 6.8±0.4/60 min respectively (Fig. 5A). Similarly, NGD 9002 administered orally (3, 10, and 30 mg/kg, −60 min, n = 8 for each dose) reduced WAS-stimulated FPO compared with vehicle+WAS group (2.6±0.6, 1.9±0.8, and 1.8±0.6 vs 3.8±0.5 number/60 min respectively (Fig. 5B). Post-hoc comparisons showed a significant reduction of fecal pellets at 30 mg/kg NGD 9002 and at 10 and 30 mg/kg NGD 98-2. In control rats (non-stressed), NGD 9002 (30 mg/kg, po) alone had no effect on FPO (Fig. 5B).

### 5. Orogastric Administration of NGD 98-2 or NGD 9002 Decreases Repeated Tonic Colorectal Distention-induced Visceral Nociceptive Hyper-responsivity

Two tonic colorectal distensions (60 mmHg for 10 min with a 30 min interval) increased the VMR monitored by increased EMG activity (Fig. 6A–B). A representative trace of the abdominal contraction response to tonic distensions in vehicle or NGD 98-2 (30 mg/kg, og) pretreated rats (-40 min before 1^st^ CRD) is shown in Fig. 6A and 6B respectively. The mean percent difference between the 1^st^ and 2^nd^ responses in the vehicle-pretreated rats was significantly higher than the mean percent difference in NGD 98-2-pretreated rats (60.4±33.4% vs −38.2±19.2%, p<0.05, n = 8-10/group, Fig. 6C). In addition, while 5 out of 8 vehicle-pretreated rats (62.5%) had at least a 10% higher response to the 2^nd^ distention when compared to the 1^st^, only 1 out of 10 rats (10%) pretreated with NGD 98-2 *(*30 mg/kg) had a 10% or higher response to the 2^nd^ CRD when compared to the 1^st^.

In a separate study where the effect of NGD 9002 was evaluated in a larger number of rats (n = 8–22/group), repeated CRD resulted in a significantly increased 2^nd^ CRD response (p<0.05, Fig. 7A & 7C). As in the NGD 98-2 experiment, the mean percent difference between the 1^st^ and 2^nd^ responses in the vehicle-pretreated rats (n = 22) was significantly higher than the mean percent difference of NGD 9002 at 30 mg/kg group (n = 17) (Fig. 7C). In addition, while 9 out of 22 vehicle pre-treated rats (41%) had at least a 10% higher response to the 2^nd^ CRD, when compared to the 1^st^, none of the 8 rats pretreated with NGD 9002 at 10 mg/kg and only 2 out of 17 of the NGD 9002 at 30 mg/kg had a 10% or higher response to the 2^nd^ CRD compared with the 1^st^ (Fig. 7A-C).

## Discussion

NGD 98-2 and NGD 9002 were identified as pre-clinical development candidates from drug discovery efforts spanning several series of topology 2 CRF_1_ antagonists. These compounds display high affinity (less than 10 nM) to both human and rat CRF_1_ receptors (Ki = 1.0 and 9.8 nM for NGD 98-2 and Ki = 2.3 and 4.3 nM for NGD 9002 respectively) and demonstrate oral efficacy [24], [25]. The present study shows that NGD 98-2 and NGD 9002, administered orogastrically, dampen centrally or peripherally injected CRF- or acute WAS-induced colonic motor stimulation (defecation) and repeated tonic CRD-induced visceral sensitization in rats. These data indicate the effectiveness of orally administered NGD 98-2 and NGD 9002 against exogenous CRF and endogenous CRF released by acute WAS [27], [35]-induced IBS-D-like symptoms in rats.

CRF injected icv at a dose of 10 µg/kg produced a significant and reproducible increase in FPO and incidence of diarrhea in 50% of rats. This is consistent with previous reports showing that icv CRF-induced enhanced colonic secretory and motor function [27], [28], [40]. Orogastric administration of the selective CRF_1_ antagonists, NGD 98-2 [24] or NGD 9002 [25] dose-dependently reduced the icv CRF-induced FPO. NGD 98-2 also dose-dependently prevented the incidence of diarrhea induced by icv CRF while NGD 9002 showed a non-significant trend. Previous studies in rats showed that icv injection of the CRF_1_ antagonist, NBI-27914 blocked icv CRF-induced defecation [28], [41]. The present data provide the first evidence that an orally administered CRF_1_ antagonist abolished icv CRF induced stimulation of both colonic propulsive motor function and diarrhea. This supports the efficacy of orally administered new generation CRF_1_ antagonists against centrally mediated CRF stimulatory effects on colonic secretory-motor function. We have recently reported that NGD 98-2 crosses the blood brain barrier upon orogastric administration to block icv CRF mediated increased locomotor activity in rats [24]. The present data extend these findings to the gastrointestinal tract. In addition, the fact that og administration of NGD 9002 has an *in vivo* IC_50_ value of 4.3 mg/kg compared to 15.7 mg/kg for NGD 98-2 in blocking icv CRF-induced defecation, while both NGDs have similar Ki to rat CRF_1_ [24], [25] suggests that NGD 9002 and NGD 98-2 may have ability to cross the blood brain barrier to block exogenous CRF action in the brain. This is of significance because most of the selective CRF_1_ antagonists used in prior studies to address the role of CRF_1_ in central CRF-induced colonic stimulation had to be administered through systemic or central injections due to their poor oral bioavailability [28], [31], [41]. The few CRF_1_ antagonists that show oral bioavailability were tested with either peripheral injection of CRF [34] or mainly in the context of icv CRF-induced behavioral outcomes such as increased locomotion, addiction, depression and anxiety [10], [22], [24]. The single double-blind placebo controlled clinical trial that evaluated the effect of an orally-administered CRF_1_ antagonist, BMS-562086 on gastrointestinal outcomes showed no improvement in colonic transit in IBS-D predominant patients [15]. The current study is the first to demonstrate the efficacy of orally administered selective non-peptide CRF_1_ antagonists to alleviate central CRF-induced colonic stimulation in rats. Of note, most of the CRF_1_ antagonists tested in prior studies had limitations for clinical use in part due to their high lipophilicity [22], [42].

Next we tested whether systemic or orogastric administration of NGD 9002 and NGD 98-2 would influence the colonic response to peripherally injected CRF. Intraperitoneal injection of CRF is well-established to act through distinct mechanisms than those initiated in the brain by central injection of CRF [43]. CRF injected ip or locally induces a direct activation of colonic cholinergic myenteric neurons, stimulates colonic 5-HT release and activates mast cells [44], [45]. NGD 98-2 injected sc at 30 mg/kg unlike lower doses (10 or 3 mg/kg) reduced defecation induced by ip CRF. Additionally NGD 9002 given orally at 30 mg/kg reduced icv CRF-induced defecation and showed a non-significant trend to reduce the incidence of diarrhea induced by ip CRF. Under the same conditions, the peptide CRF_1_/CRF_2_ antagonist astressin [46] injected ip at 30 µg/kg completely prevented both the defecation and diarrhea as previously reported [31]. Consistent with these findings, the CRF_1_ antagonist NBI 35965 injected sc or og (10–20 mg/kg) reduced ip or intravenous CRF induced stimulation of distal colonic transit in rats [34]. The present data are also in line with reported preventive action of other CRF_1_ antagonists such as CP-154,526 given sc against ip CRF or the selective CRF_1_ agonist, cortagine,-induced stimulation of colonic motor function [31], [47].

The effects of central and peripheral CRF on the colon have long been suggested to mimic those produced by acute stressors [48]. In particular, acute exposure to WAS produced a reliable and significant increase in FPO which we demonstrated previously to involve activation of CRF_1_ receptors located in both the brain and the colon [6]. In the present study, orogastric administration of NGD 9002 and NGD 98-2, while having no effect on defecation in a non-stressed rat, effectively reduced the enhanced colonic motor response caused by WAS. To date, with the exception of one study that tested the efficacy of an orogastrically-administered CRF_1_ antagonist, NBI 35965 on defecation induced by WAS [34], the overwhelming majority of studies on acute stress-induced colonic motor stimulation have used sc injections of the non-peptide selective CRF_1_ antagonist CP-154,524 [31], [49]. The present study adds, to the orally-active selective CRF_1_ antagonists, two novel compounds for use in preclinical and potential clinical studies on gut motor response to stress.

Lastly, while non-selective and selective CRF_1_ antagonists have been well documented to prevent stress- or icv or ip CRF-induced visceral sensitization in rodents [34], [47], [50]–[53], the effects of orally-active CRF_1_ antagonists against nociceptive hyper-responsivity to tonic noxious colonic stimulation have not been studied. Repeated tonic CRD-induced visceral hypersensitivity has been established in rats [29], [37], [54], mice [55], and humans [56], [57]. In rats, we previously reported that such a hypersensitization is not associated with colonic lesion and is prevented by the ip injection of a selective CRF_2_ receptor agonist, urocortin-2 [37], a sc injection of a selective CRF_1_ antagonist antalarmin [29] or by oral administration of pregabalin, a ligand to the α_2_δ subunit of the voltage-gated calcium channel [58]. In the present study, repeated tonic CRD resulted in an enhanced VMR to the 2^nd^ distention compared to the 1^st^, indicating the development of acute hypersensitivity to a noxious visceral stimuli. This response is prevented by oral-administration of NGD 98-2 and NGD 9002, indicative of a role of CRF_1_ in the response. The demonstration of the involvement of CRF_1_ in repeated mechanical CRD-induced nociceptive hyper-responsivity is in line with previous studies using sc antalarmin [29].

The exact site(s) and mechanism(s) of action of the orally-active NGD compounds cannot be determined from the present study. Tonic CRD is shown to activate ERK phosphorylation in the lumbosacral spinal segments in rats and the response is blunted by CRF_2_ activation [37]. Spinal ERK phosphorylation modulates neuronal excitability and plays an important role in hyperalgesia after noxious somatic stimuli and inflammation [59] suggesting a possible sensitization occurring during repeated CRD as well. On the other hand, activation of peripheral CRF_1_ receptors by the CRF_1_ selective peptide agonist, cortagine, causes visceral hyperalgesia in rats [47] suggesting a possible peripheral action of orally-administered NGD compounds to prevent repeated tonic CRD-induced sensitization. Taken together, given that both central and peripheral sensitizations are reported to occur during visceral hyperalgesia [60] and the fact that the orally-administered NGD 98-2 crosses the blood brain barrier with high brain exposure leading to CRF_1_ receptor occupancy assessed by autoradiography [24], it is likely that the compounds block CRF_1_-mediated central and peripheral visceral hyper responsiveness.

In summary, significant progress has been made in the design and development of non- peptide CRF_1_ receptor antagonists [22], [61]. However, high lipophilic characteristics and potential toxicity have hampered their translational applications [22], [42], [61]. Thus, of the numerous non-peptide small molecule CRF_1_ antagonists developed, to date very few have moved forward to clinical trials [13], [61]. NGD 98-2 and NGD 9002 are topology 2 CRF_1_ antagonists with high affinity to CRF_1_, high oral bioavailablity and low lypophilicity. Orally-administered NGD 98-2 and NGD 9002 effectively prevented icv CRF-induced activation of defecation in rats. The present data show also that the selective CRF_1_ antagonists blunt WAS-induced colonic motor activation and prevented repeated CRD-induced visceral hypersensitivity. The data strengthen the concept that activation of CRF_1_ signaling pathway plays a role in mediating acute stress-related stimulation of colonic motor function as well as visceral sensitization to CRD in healthy rats. Although, several preclinical studies have shown, early on, the role of CRF_1_ receptors in stress-related colonic stimulation and suggested a therapeutic potential of CRF_1_ antagonists against stress-related diarrhea and visceral hypersensitivity [7], [40], [50], the report that a CRF_1_ antagonist lacks efficacy to influence intestinal transit and diarrhea in IBS patients [15], raises concern on the potential use of CRF_1_ antagonists to alleviate symptoms in IBS patients. Several reasons including the testing of CRF_1_ antagonists in preclinical studies mainly in an acute stress-context, while in clinical studies the state of acute or chronic stress of patients is not well determined; the presence of over 11 splice variants of CRF_1_ receptors in humans [62]; the existence of differences in binding kinetics, association and dissociation rate and efficacy of CRF_1_ antagonists used [61], [63] may account for the discrepancy. There is also a lack of information, in previous clinical studies, on whether the antagonist regimens used is optimum to block both central and peripheral CRF_1_ receptors. In this context, the development of new CRF_1_ antagonists such as NGD 98-2 and NGD 9002 that have better oral bioavailability opens new venues to understand the potential role and mechanisms of CRF_1_ receptors in stress-sensitive functional bowel disorders such as IBS.

## References

[1] Vale W, Spiess J, Rivier C, Rivier J (1981) Characterization of a 41-residue ovine hypothalamic peptide that stimulates secretion of corticotropin and b-endorphin. Science 213: 1394–1397. 6267699 10.1126/science.6267699

[2] Bale TL, Vale WW (2004) CRF and CRF receptor: Role in stress responsivity and other behaviors. Annu Rev Pharmacol Toxicol 44: 525–557. 14744257 10.1146/annurev.pharmtox.44.101802.121410

[3] Hauger RL, Grigoriadis DE, Dallman MF, Plotsky PM, Vale WW, et al (2003) International Union of Pharmacology. XXXVI. Current Status of the Nomenclature for Receptors for Corticotropin-Releasing Factor and Their Ligands. Pharmacol Rev 55: 21–26. 12615952 10.1124/pr.55.1.3

[4] Farrokhi CB, Tovote P, Blanchard RJ, Blanchard DC, Litvin Y, et al (2007) Cortagine: behavioral and autonomic function of the selective CRF receptor subtype 1 agonist. CNS Drug Rev 13: 423–443. 18078427 10.1111/j.1527-3458.2007.00027.x PMC6494149

[5] Leonard BE (2005) The HPA and immune axes in stress: the involvement of the serotonergic system. Eur Psychiatry 20 Suppl 3S302–S306. 16459240 10.1016/s0924-9338(05)80180-4

[6] Stengel A, Taché Y (2010) Corticotropin-releasing factor signaling and visceral response to stress. Exp Biol Med (Maywood ) 235: 1168–1178. 20881321 10.1258/ebm.2010.009347 PMC3169435

[7] Taché Y, Bonaz B (2007) Corticotropin-releasing factor receptors and stress-related alterations of gut motor function. J Clin Invest 117: 33–40. 17200704 10.1172/JCI30085 PMC1716215

[8] Larauche M, Mulak A, Taché Y (2011) Stress-related alterations of visceral sensation: animal models for irritable bowel syndrome study. J Neurogastroenterol Motil 17: 213–234. 21860814 10.5056/jnm.2011.17.3.213 PMC3155058

[9] Taché Y, Brunnhuber S (2008) From Hans Selye’s discovery of biological stress to the identification of corticotropin-releasing factor signaling pathways: implication in stress-related functional bowel diseases. Ann N Y Acad Sci 1148: 29–41. 19120089 10.1196/annals.1410.007 PMC2993154

[10] Zorrilla EP, Heilig M, de Wit H, Shaham Y (2013) Behavioral, biological, and chemical perspectives on targeting CRF(1) receptor antagonists to treat alcoholism. Drug Alcohol Depend 128: 175–186. 23294766 10.1016/j.drugalcdep.2012.12.017 PMC3596012

[11] Ising M, Zimmermann US, Kunzel HE, Uhr M, Foster AC, et al (2007) High-affinity CRF_1_ receptor antagonist NBI-34041: preclinical and clinical data suggest safety and efficacy in attenuating elevated stress response. Neuropsychopharmacology 32: 1941–1949. 17287823 10.1038/sj.npp.1301328

[12] Sagami Y, Shimada Y, Tayama J, Nomura T, Satake M, et al (2004) Effect of a corticotropin releasing hormone receptor antagonist on colonic sensory and motor function in patients with irritable bowel syndrome. Gut 53: 958–964. 15194643 10.1136/gut.2003.018911 PMC1774093

[13] Paez-Pereda M, Hausch F, Holsboer F (2011) Corticotropin releasing factor receptor antagonists for major depressive disorder. Expert Opin Investig Drugs 20: 519–535. 10.1517/13543784.2011.565330 21395482

[14] Hubbard CS, Labus JS, Bueller J, Stains J, Suyenobu B, et al (2011) Corticotropin-releasing factor receptor 1 antagonist alters regional activation and effective connectivity in an emotional-arousal circuit during expectation of abdominal pain. J Neurosci 31: 12491–12500. 21880911 10.1523/JNEUROSCI.1860-11.2011 PMC3399687

[15] Sweetser S, Camilleri M, Linker Nord SJ, Burton DD, Castenada L, et al (2009) Do Corticotropin Releasing Factor-1 Receptors Influence Colonic Transit and Bowel Function in Females with Irritable Bowel Syndrome? Am J Physiol Gastrointest Liver Physiol. 296: G1299–306. 10.1152/ajpgi.00011.2009 PMC2697942 19342506

[16] Zobel AW, Nickel T, Kunzel HE, Ackl N, Sonntag A, et al (2000) Effects of the high-affinity corticotropin-releasing hormone receptor 1 antagonist R121919 in major depression: the first 20 patients treated. J Psychiatr Res 34: 171–181. 10867111 10.1016/s0022-3956(00)00016-9

[17] Bailey JE, Papadopoulos A, Diaper A, Phillips S, Schmidt M, et al (2011) Preliminary evidence of anxiolytic effects of the CRF(1) receptor antagonist R317573 in the 7.5% CO(2) proof-of-concept experimental model of human anxiety. J Psychopharmacol 25: 1199–1206. 21555331 10.1177/0269881111400650

[18] Binneman B, Feltner D, Kolluri S, Shi Y, Qiu R, et al (2008) A 6-week randomized, placebo-controlled trial of CP-316,311 (a selective CRH1 antagonist) in the treatment of major depression. Am J Psychiatry 165: 617–620. 18413705 10.1176/appi.ajp.2008.07071199

[19] Coric V, Feldman HH, Oren DA, Shekhar A, Pultz J, et al (2010) Multicenter, randomized, double-blind, active comparator and placebo-controlled trial of a corticotropin-releasing factor receptor-1 antagonist in generalized anxiety disorder. Depress Anxiety 27: 417–425. 20455246 10.1002/da.20695

[20] Schulz DW, Mansbach RS, Sprouse J, Braselton JP, Collins J, et al (1996) CP-154,526: a potent and selective nonpeptide antagonist of corticotropin releasing factor receptors. Proc Natl Acad Sci U S A 93: 10477–10482. 8816826 10.1073/pnas.93.19.10477 PMC38410

[21] Griebel G, Simiand J, Steinberg R, Jung M, Gully D, et al (2002) 4-(2-Chloro-4-methoxy-5-methylphenyl)-N-[(1S)-2-cyclopropyl-1-(3-fluoro-4- methylphenyl)ethyl]5-methyl-N-(2-propynyl)-1, 3-thiazol-2-amine hydrochloride (SSR125543A), a potent and selective corticotrophin-releasing factor(1) receptor antagonist. II. Characterization in rodent models of stress-related disorders. J Pharmacol Exp Ther 301: 333–345. 11907191 10.1124/jpet.301.1.333

[22] Kehne J, De Lombaert S (2002) Non-peptidic CRF_1_ receptor antagonists for the treatment of anxiety, depression and stress disorders. Curr Drug Targets CNS Neurol Disord 1: 467–493. 12769601 10.2174/1568007023339049

[23] Chen C (2006) Recent advances in small molecule antagonists of the corticotropin-releasing factor type-1 receptor-focus on pharmacology and pharmacokinetics. Curr Med Chem 13: 1261–1282. 16712469 10.2174/092986706776873014

[24] Hodgetts KJ, Ge P, Yoon T, De Lombaert S, Brodbeck R, et al (2011) Discovery of N-(1-ethylpropyl)-[3-methoxy-5-(2-methoxy-4-trifluoromethoxyphenyl)-6-methyl-pyra zin-2-yl]amine 59 (NGD 98–2): an orally active corticotropin releasing factor-1 (CRF-1) receptor antagonist. J Med Chem 54: 4187–4206. 21618986 10.1021/jm200365y

[25] Yoon T, Ge P, Delombaert S, Horvath R (3–4-2004) 5-substituted-2-arylpyrazines as modulators of CRF receptors. Patent: International Application Published Under The Patent Operation Treaty (PCT). 127 p.

[26] Saunders PR, Maillot C, Million M, Taché’ Y (2002) Peripheral corticotropin-releasing factor induces diarrhea in rats: role of CRF_1_ receptor in fecal watery excretion. Eur J Pharmacol 435: 231–235. 11821031 10.1016/s0014-2999(01)01574-6

[27] Taché Y, Martinez V, Wang L, Million M (2004) CRF_1_ receptor signaling pathways are involved in stress-related alterations of colonic function and viscerosensitivity: implications for irritable bowel syndrome. Br J Pharmacol 141: 1321–1330. 15100165 10.1038/sj.bjp.0705760 PMC1574904

[28] Martinez V, Taché Y (2001) Role of CRF receptor 1 in central CRF-induced stimulation of colonic propulsion in rats. Brain Res 893: 29–35. 11222989 10.1016/s0006-8993(00)03277-7

[29] Million M, Maillot C, Adelson DW, Nozu T, Gauthier A, et al (2005) Peripheral injection of sauvagine prevents repeated colorectal distention-induced visceral pain in female rats. Peptides 26: 1188–1195. 15949637 10.1016/j.peptides.2005.02.004

[30] Seymour PA, Schmidt AW, Schulz DW (2003) The pharmacology of CP-154,526, a non-peptide antagonist of the CRH1 receptor: a review. CNS Drug Rev 9: 57–96. 12595912 10.1111/j.1527-3458.2003.tb00244.x PMC6741649

[31] Maillot C, Million M, Wei JY, Gauthier A, Taché Y (2000) Peripheral corticotropin-releasing factor and stress-stimulated colonic motor activity involve type 1 receptor in rats. Gastroenterology 119: 1569–1579. 11113078 10.1053/gast.2000.20251

[32] Martinez V, Rivier J, Wang L, Taché Y (1997) Central injection of a new corticotropin-releasing factor (CRF) antagonist, astressin, blocks CRF- and stress-related alterations of gastric and colonic motor function. J Pharmacol Exp Ther 280: 754–760. 9023288

[33] Paxinos G, Watson C (1998) The rat brain in stereotaxic coordinates. Orlando: Academic Press. 116 p.

[34] Million M, Grigoriadis DE, Sullivan S, Crowe PD, McRoberts JA, et al (2003) A novel water-soluble selective CRF_1_ receptor antagonist, NBI 35965, blunts stress-induced visceral hyperalgesia and colonic motor function in rats. Brain Res 985: 32–42. 12957366 10.1016/s0006-8993(03)03027-0

[35] Bonaz B, Taché Y (1994) Water-avoidance stress-induced c-fos expression in the rat brain and stimulation of fecal output: role of corticotropin-releasing factor. Brain Res 641: 21–28. 8019847 10.1016/0006-8993(94)91810-4

[36] Ness TJ, Gebhart GF (1988) Colorectal distension as a noxious visceral stimulus: physiologic and pharmacologic characterization of pseudoaffective reflexes in the rat. Brain Res 450: 153–169. 3401708 10.1016/0006-8993(88)91555-7

[37] Million M, Wang L, Wang Y, Adelson DW, Yuan PQ, et al (2006) CRF2 receptor activation prevents colorectal distension-induced visceral pain and spinal ERK1/2 phosphorylation in rats. Gut 55: 172–181. 15985561 10.1136/gut.2004.051391 PMC1856510

[38] Coutinho SV, Plotsky PM, Sablad M, Miller JC, Zhou H, et al (2002) Neonatal maternal separation alters stress-induced responses to viscerosomatic nociceptive stimuli in rat. Am J Physiol Gastrointest Liver Physiol 282: G307–G316. 11804852 10.1152/ajpgi.00240.2001

[39] Miampamba M, Maillot C, Million M, Taché Y (2002) Peripheral CRF activates myenteric neurons in the proximal colon through CRF_1_ receptor in conscious rats_._ . Am J Physiol Gastrointest Liver Physiol 282: G857–G865. 11960782 10.1152/ajpgi.00434.2001

[40] Williams CL, Peterson JM, Villar RG, Burks TF (1987) Corticotropin-releasing factor directly mediates colonic responses to stress. Am J Physiol 253: G582–G586. 2821826 10.1152/ajpgi.1987.253.4.G582

[41] Ataka K, Kuge T, Fujino K, Takahashi T, Fujimiya M (2007) Wood creosote prevents CRF-induced motility via 5-HT3 receptors in proximal and 5-HT4 receptors in distal colon in rats. Auton Neurosci 133: 136–145. 17182287 10.1016/j.autneu.2006.11.002

[42] Kehne JH, Cain CK (2010) Therapeutic utility of non-peptidic CRF_1_ receptor antagonists in anxiety, depression, and stress-related disorders: evidence from animal models. Pharmacol Ther 128: 460–487. 20826181 10.1016/j.pharmthera.2010.08.011 PMC3373002

[43] Stengel A, Taché Y (2009) Neuroendocrine control of the gut during stress: corticotropin-releasing factor signaling pathways in the spotlight. Annu Rev Physiol 71: 219–239. 18928406 10.1146/annurev.physiol.010908.163221 PMC2714186

[44] Kimura T, Amano T, Uehara H, Ariga H, Ishida T, et al (2007) Urocortin I is present in the enteric nervous system and exerts an excitatory effect via cholinergic and serotonergic pathways in the rat colon. Am J Physiol Gastrointest Liver Physiol 293: G903–G910. 17717045 10.1152/ajpgi.00066.2007

[45] Overman EL, Rivier JE, Moeser AJ (2012) CRF induces intestinal epithelial barrier injury via the release of mast cell proteases and TNF-alpha. PLoS One 7: e39935. 22768175 10.1371/journal.pone.0039935 PMC3386952

[46] Gulyas J, Rivier C, Perrin M, Koerber SC, Sutton S, et al (1995) Potent, structurally constrained agonists and competitive antagonists of corticotropin-releasing factor. Proc Natl Acad Sci USA 92: 10575–10579. 7479843 10.1073/pnas.92.23.10575 PMC40654

[47] Larauche M, Gourcerol G, Wang L, Pambukchian K, Brunnhuber S, et al (2009) Cortagine, a CRF_1_ agonist, induces stress-like alterations of colonic function and visceral hypersensitivity in rodents primarily through peripheral pathways. Am J Physiol Gastrointest Liver Physiol 297: G215–G227. 19407218 10.1152/ajpgi.00072.2009 PMC2711753

[48] Taché Y, Million M (2006) Central corticotropin-releasing factor and the hypothalamic-pituitary-adrenal axis in gastrointestinal physiology. In: Johnson LR, Wood J, editors. Physiology of the Gastrointestinal Tract. Academic Press. 791–816.

[49] Martinez V, Taché Y (2006) CRF_1_ receptors as a therapeutic target for irritable bowel syndrome. Curr Pharm Des 12: 1–18. 10.2174/138161206778743637 17100612

[50] Gué M, Del Rio-Lacheze C, Eutamene H, Theodorou V, Fioramonti J, et al (1997) Stress-induced visceral hypersensitivity to rectal distension in rats: role of CRF and mast cells. Neurogastroenterol Motil 9: 271–279. 9430796 10.1046/j.1365-2982.1997.d01-63.x

[51] Greenwood-Van Meerveld B, Johnson AC, Cochrane S, Schulkin J, Myers DA (2005) Corticotropin-releasing factor 1 receptor-mediated mechanisms inhibit colonic hypersensitivity in rats. Neurogastroenterol Motil 17: 415–422. 15916629 10.1111/j.1365-2982.2005.00648.x

[52] Schwetz I, McRoberts JA, Coutinho SV, Bradesi S, Gale G, et al (2005) Corticotropin-releasing factor receptor 1 mediates acute and delayed stress-induced visceral hyperalgesia in maternally separated Long-Evans rats. Am J Physiol Gastrointest Liver Physiol 289: G704–G712. 15994424 10.1152/ajpgi.00498.2004

[53] Larauche MH, Bradesi S, Million M, McLean P, Taché YF, et al (2008) Corticotropin releasing factor type 1 receptors mediate the visceral hyperalgesia induced by repeated psychological stress in rats. Am J Physiol Gastrointest Liver Physiol. 294: G1033–40. 10.1152/ajpgi.00507.2007 18308857

[54] Gaudreau GA, Plourde V (2004) Involvement of N-methyl-d-aspartate (NMDA) receptors in a rat model of visceral hypersensitivity. Behav Brain Res 150: 185–189. 15033291 10.1016/j.bbr.2003.07.004

[55] Larsson M, Arvidsson S, Ekman C, Bayati A (2003) A model for chronic quantitative studies of colorectal sensitivity using balloon distension in conscious mice – effects of opioid receptor agonists. Neurogastroenterol Motil 15: 371–381. 12846725 10.1046/j.1365-2982.2003.00418.x

[56] Bouin M, Plourde V, Boivin M, Riberdy M, Lupien F, et al (2002) Rectal distention testing in patients with irritable bowel syndrome: sensitivity, specificity, and predictive values of pain sensory thresholds. Gastroenterology 122: 1771–1777. 12055583 10.1053/gast.2002.33601

[57] Munakata J, Naliboff B, Harraf F, Kodner A, Lembo T, et al (1997) Repetitive sigmoid stimulation induces rectal hyperalgesia in patients with irritable bowel syndrome. Gastroenterology 112: 55–63. 8978343 10.1016/s0016-5085(97)70219-1

[58] Million M, Wang L, Adelson DW, Roman F, Diop L, et al (2007) Pregabalin decreases visceral pain and prevents spinal neuronal activation in rats. Gut 56: 1482–1484. 17872585 10.1136/gut.2007.129304 PMC2000255

[59] Hu HJ, Gereau RW (2003) ERK integrates PKA and PKC signaling in superficial dorsal horn neurons. II. Modulation of neuronal excitability. J Neurophysiol 2003 Sep; 90 (3): 1680–8 Epub 2003 May 15 90: 1680–1688. 10.1152/jn.00341.2003 12750418

[60] Sengupta JN (2009) Visceral pain: the neurophysiological mechanism. Handb Exp Pharmacol 31–74. 10.1007/978-3-540-79090-7_2 PMC3156094 19655104

[61] Zorrilla EP, Koob GF (2010) Progress in corticotropin-releasing factor-1 antagonist development. Drug Discov Today 15: 371–383. 20206287 10.1016/j.drudis.2010.02.011 PMC2864802

[62] Zmijewski MA, Slominski AT (2010) Emerging role of alternative splicing of CRF_1_ receptor in CRF signaling. Acta Biochim Pol 57: 1–13. 20234885 PMC2883312

[63] Fleck BA, Hoare SR, Pick RR, Bradbury MJ, Grigoriadis DE (2012) Binding kinetics redefine the antagonist pharmacology of the corticotropin-releasing factor type 1 receptor. J Pharmacol Exp Ther 341: 518–531. 22357972 10.1124/jpet.111.188714

